# Forensic Applications of Microbiomics: A Review

**DOI:** 10.3389/fmicb.2020.608101

**Published:** 2021-01-13

**Authors:** Jake M. Robinson, Zohar Pasternak, Christopher E. Mason, Eran Elhaik

**Affiliations:** ^1^Department of Landscape, University of Sheffield, Sheffield, United Kingdom; ^2^Healthy Urban Microbiome Initiative (HUMI), Adelaide, SA, Australia; ^3^Quality Assurance and Evidence Unit, Division of Identification and Forensic Science (DIFS), National Headquarters of the Israel Police, Jerusalem, Israel; ^4^Department of Physiology and Biophysics, Weill Cornell Medicine, New York, NY, United States; ^5^The HRH Prince Alwaleed Bin Talal Bin Abdulaziz Alsaud Institute for Computational Biomedicine, Weill Cornell Medicine, New York, NY, United States; ^6^The WorldQuant Initiative for Quantitative Prediction, Weill Cornell Medicine, New York, NY, United States; ^7^Department of Biology, Lund University, Lund, Sweden

**Keywords:** microbiome, forensic microbiology, forensic science, microbial forensics, metagenomics, postmortem interval, microbiomics

## Abstract

The rise of microbiomics and metagenomics has been driven by advances in genomic sequencing technology, improved microbial sampling methods, and fast-evolving approaches in bioinformatics. Humans are a host to diverse microbial communities in and on their bodies, which continuously interact with and alter the surrounding environments. Since information relating to these interactions can be extracted by analyzing human and environmental microbial profiles, they have the potential to be relevant to forensics. In this review, we analyzed over 100 papers describing forensic microbiome applications with emphasis on geolocation, personal identification, trace evidence, manner and cause of death, and inference of the postmortem interval (PMI). We found that although the field is in its infancy, utilizing microbiome and metagenome signatures has the potential to enhance the forensic toolkit. However, many of the studies suffer from limited sample sizes and model accuracies, and unrealistic environmental settings, leaving the full potential of microbiomics to forensics unexplored. It is unlikely that the information that can currently be elucidated from microbiomics can be used by law enforcement. Nonetheless, the research to overcome these challenges is ongoing, and it is foreseeable that microbiome-based evidence could contribute to forensic investigations in the future.

## Introduction

For over 100 years, microbiology has played a relatively diminutive role in forensic science (MacCallum and Hastings, 1899). In the early 1990s, the sequencing of amplified viral DNA was used to support a case alleging the transmission of Human Immunodeficiency Virus from a dentist to several patients in Florida, United States (Smith and Waterman, 1992). The emergence of PCR-mediated genotyping of bacteria was considered to be a valuable forthcoming tool in forensics—e.g., van Belkum (1994) suggested that forensic science would soon be a major area for the application of PCR-mediated genotyping due to the rapidity of technological advances at the time (van Belkum, 1994). In the mid-1990s, fungal and pollen spore analyses were also developed, allowing investigators to differentiate between soil types, which in turn allowed linking substrate items to particular sites (Bruce and Dettmann, 1996; Bryant and Mildenhall, 1998). However, it was not until the early 2000s and the rise of bioterrorism that *microbial forensics*—the “scientific discipline dedicated to analyzing evidence from a bioterrorism act, biocrime, or inadvertent microorganism/toxin release for attribution purposes”—emerged in response to the new threat (Budowle et al., 2003; Carter et al., 2017).

Many forensic applications have been limited to individual taxa analyses, and *microbial forensics* has, historically, been constrained by a lack of available and cost-effective sequencing technologies (Berglund et al., 2011; Kuiper, 2016). This approach has changed dramatically in the last decade as advances in genomic sequencing technology, and new methods for processing complex community datasets (and often low biomass samples) have led to the advent of a new field of microbiomics. The science and study of the microbiome (Statnikov et al., 2013; Capasso et al., 2019) combined with metagenomics (all genomes from a sample) have enhanced the development of the microbial forensic toolkit (Clarke et al., 2017; Hampton-Marcell et al., 2017).

As of March 2019, the conviction rate for homicides in England and Wales (United Kingdom) was only 79% (Office for National Statistics, 2019), slightly higher than in the US (∼70%) (Bureau for Justice Statistics., 2019). Across the globe, there is also a high prevalence of wrongful convictions and often insufficient evidence to convict a perpetrator of a crime (Sangero and Halpert, 2007; Ingemann-Hansen et al., 2008; LaPorte, 2017; Walsh et al., 2017). According to the Innocence Project, a national litigation and public policy organization dedicated to exonerating wrongfully convicted individuals, to date, 375 people in the United States have been exonerated by DNA testing, including 21 who served time on death row (Innocent Project, 2020). There is thereby a strong interest from the public, lawmakers, and the law enforcement system to augment and expand the forensic toolkit, including molecular methods. Microorganisms are abundant in and on the human body (microbial cells can outnumber or equal the total number of human somatic cells) (Noel et al., 2014; Sender et al., 2016; Vázquez-Baeza et al., 2018), in surrounding environments, and on objects associated with a crime (Desmond et al., 2018; Oliveira and Amorim, 2018). A growing body of evidence suggests that forensically relevant microbial profiles could be used as evidence or, at the very least, complement traditional investigative methods (Metcalf et al., 2017; Schmedes et al., 2017; Richardson et al., 2019; Phan et al., 2020). This use of microbial profiles as evidence is done using computational tools that are being developed alongside new approaches in bioinformatics, processing tools, and refined protocols. However, since the field is still in its infancy (Goudarzi et al., 2016; Komaroff, 2018) and historically underfunded (Morgan and Levin, 2019), there is much uncertainty as to the true potential of microbiomic tools in forensics.

**FIGURE 1:**
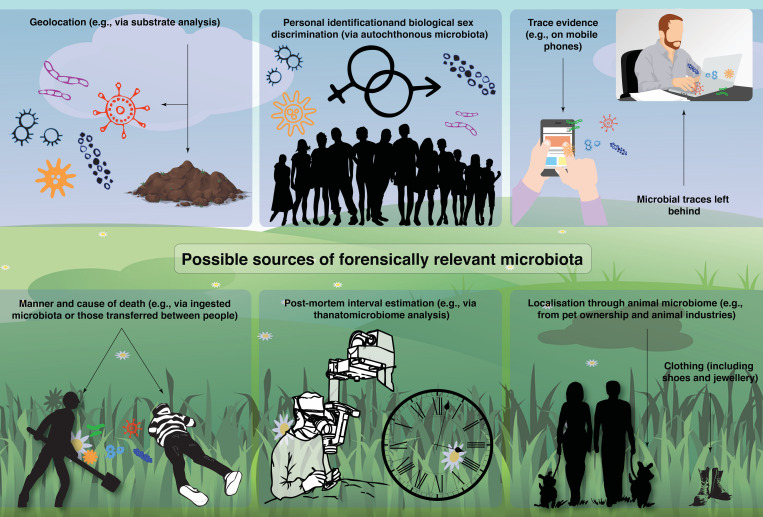
A summary of possible sources of forensically relevant microbiota identified by the literature review.

In this review, we provide an overview of past, current, and future potential applications of microbiomics in forensics. Specifically, we will discuss the six most comprehensively researched themes (Figure 1): including geolocation (e.g., substrate analysis and different spatial dimensions and the power of machine learning), personal identification, biological sex determination, trace evidence, manner and cause of death (e.g., death by drowning), PMI, and other applications (e.g., localization through animal microbiomes).

## Major Themes in the Forensic Microbiology Literature

### Geolocation

In the past few years, intensive work has been carried out to characterize environmental microbiome, particularly in urban environments and transit systems. These studies have demonstrated that unique community profiles may exist in certain areas of a city (Afshinnekoo et al., 2015; Rosenfeld et al., 2016), as well as “molecular echoes” of environmental events, and even a forensic capacity for geospatial microbiomic data (MetaSUB International Consortium, 2016; Danko et al., 2019a). In the following, we focus on two leading aspects of geolocalization.

#### Substrate Analysis

The potential of analyzing microbial profiles from the soil is increasingly being recognized in forensic microbiological research. Both the rhizosphere and bulk soil microbiomes exhibit a high level of heterogeneity between different sites. As such, with methodological refinement, soil microbiome samples could provide valuable biogeographic data to localize the origin of the soil sample. Another potential application is the acquisition of information to help determine the provenance of an item(s) associated with a crime.

Habtom et al. (2019) demonstrated distance-decay relationships between microbial samples from the soil at a local scale (25–1,000 m) (*n* = 5 sites, *n* = 2–4 soil types, and five replications). The results showed that the greater the distance between the samples, the more they differed, suggesting that both soil type and geographic location are important factors in determining microbial community composition. Indeed, patch discrimination using the soil microbiome has previously been demonstrated (Macdonald et al., 2011), and Jesmok et al. (2016) correctly classified 95.4% of soil bacterial profiles to their location of origin using various methods including abundance charts, non-metric multidimensional scaling, analysis of similarity, and *k*-nearest neighbor. However, this was a feasibility study with a modest sample size (*n* = 19). Further studies with larger sample sizes and replications are needed to explore the full potential of this approach.

Evaluating the microbial communities in an assemblage of soil samples (e.g., soils from a crime scene or alibi site and other intermediary sites) could be useful in forensics. Samples originating from a mixture of different soil substrates have been correctly differentiated by using a combination of Ribosomal Intergenic Spacer Analysis and 16S rRNA gene sequencing (Demanèche et al., 2017). Recent evidence suggests that 18S rRNA gene sequencing can also provide greater discriminatory power over traditional Mid-Infrared spectroscopy at fine scales for eukaryotic species (Young et al., 2015). Furthermore, Sanachai et al. (2016) demonstrated that the site origin of soil, obtained from the sole of a shoe, could be elucidated by comparing the similarity of soil bacterial 16S rDNA profiles acquired by the denaturing gradient gel electrophoresis technique.

Despite the potential in this field, further limitations need to be identified and addressed. For example, Pasternak et al. (2019) identified several potentially limiting factors to consider when interpreting the results of microbiomic analyses. For instance, soil samples are incredibly complex and highly heterogeneous even at short spatial scales, which presents a major issue to using these in a forensic context, and microbiomes exhibit a high level of physical, chemical, and biological diversity in both space and time. Pasternak et al. (2013) showed that actinobacterial fingerprints significantly differed between two seasons (summer and winter) at the same sites—implying that temporally-associated issues could arise. Keet et al. (2019) have also pointed out that soil microbiome composition can change as a result of abiotic soil conditions and plant community patterns—which poses a considerable challenge to the accuracy of results.

Metagenomic analysis of gravesoil and the soil around human and non-human animal cadavers has been undertaken for forensic purposes. Carter et al. (2015) investigated microbial community succession in soils associated with swine cadavers across two seasons (summer and winter). They demonstrated that postmortem microbial communities changed in specific and reproducible ways, but decomposition effects on soil microbial communities differed significantly between seasons. The authors suggest that the ecological succession of microbial communities will be useful for forensic investigations, but future research should aim to gain a greater understanding of seasonality on decomposition. The sample size for this study was not explicitly stated, but according to the ordination plots, it appears to be modest (*n* ≤ 10 per treatment). Therefore, results should be interpreted with caution.

Adserias-Garriga et al. (2017) investigated daily thanatomicrobiome changes in the soil as an approach to estimate PMI. They collected soil samples from around human cadavers (*n* = 1 male and *n* = 2 females) and demonstrated successional changes on a daily basis. Rapid growth of *Firmicutes* was observed from the bloat stage to advance decay (<5% relative abundance at day 1 to 75% relative abundance day 12), and the authors proposed a *Firmicutes* growth curve to estimate PMI. However, the authors state that the growth curve results may only apply under Tennessee summer conditions and that confirmatory research is needed using a larger number of cadavers and under different environmental conditions. The results do, however, corroborate those of Finley et al. (2016), who evaluated microbial communities associated with gravesoil human cadavers (*n* = 18). The researchers allowed the cadavers to decompose over a range of decomposition time periods (3–303 days) and showed increases in the relative abundance of *Firmicutes* in surface bodies over the decomposition period (from ∼10% at 0–3 months to ∼40% at 7–9 months).

Singh et al. (2018) investigated the spatial (0, 1, and 5 m) dynamics of human cadaver decomposition on soil bacterial community structure. They collected soil samples from each spatial buffer (*n* = 14 for the 0 m, *n* = 17 for both 1 and 5 m) and observed evidence of a predictable response to cadaver decomposition that varied over space. Bacterial community composition (beta diversity) at 0 m was significantly different from the 1 and 5 m communities, whereas there were no significant differences between the 1 and 5 m communities. The researchers also found that bacterial alpha diversity was significantly lower in the 0 m samples, suggesting that the additional nutrient input from the cadavers may reduce bacterial alpha diversity. This study provides additional spatial-compositional insights to complement the growing body of knowledge in this area of forensic microbiome applications.

Soil microbiome analysis has the potential to be used in forensics; however, additional research is required to validate the sensitivity and reproducibility of results (Young et al., 2017). Overall, to gain a greater understanding of both spatial and temporal dynamics associated with the microbiome and to develop techniques to mitigate similar pitfalls, further microbiome surveys are essential.

#### Different Spatial Dimensions and the Power of Machine Learning

The growing interest in sampling and predicting environmental microbiome profiles at different spatial scales and orientations (e.g., between households, cities, states, and altitudes) to provide information on the location and provenance of people and objects resulted in the development of a multitude of approaches. Chase et al. (2016) identified the three cities where nine offices were located with 85% accuracy based on analyzing office microbiome samples using sampling plates, and although this study suffers from a small sample size (*n* = 3 per office), it demonstrates potential with further refinement. Lax et al. (2014) analyzed samples from household occupants (*n* = 1625 from 18 participants in 10 houses) and their built environments. The authors matched feet microbiome samples to the house with 82.9% accuracy—a relatively low degree of accuracy from an evidentiary perspective but demonstrating the potential of such methods for fine-scale biolocalization. Walker and Datta (2019) analyzed whole-genome sequenced microbiota sampled from 12 cities in seven different countries as part of the 2018 CAMDA MetaSUB Forensic Challenge. The CAMDA dataset (*n* = 30) included three mystery samples. The authors applied machine learning techniques to identify the geographical provenance of the microbiome samples. Up to 90% of the samples were correctly classified, demonstrating the potential of machine learning applications to biogeography, although further evidence is necessary to employ these applications in an evidentiary context. In a related study, Ryan (2019) applied a random forest classifier built on a dataset of 311 city microbiome samples. Their method correctly classified 83.3% of the mystery samples. Grantham et al. (2019) presented a different algorithm for predicting the geolocation of fungal samples from dust (*n* = 1300) in the United States using deep neural network classifiers. Applied to a global dataset of samples from 28 countries, the authors state that their algorithms make “good point predictions” with >50% of the geolocation errors under 100 km for US-wide analysis and nearly 90% classification accuracy of a sample’s country of origin for the global analysis. This particular field, combining microbiomics and machine learning, is in its infancy, and future studies would benefit from larger sample sizes and improved classification accuracy before such approaches can be used with confidence in a forensic context.

Another important spatial factor to consider is that microbiome compositions do not only differ in horizontal space. Skin microbiomes have also been shown to differ between humans living in high and low altitudes. For example, Zeng et al. (2017) collected skin microbiome samples from humans (*n* = 99) and pigs (*n* = 82) in Tibet. They found enrichments of several bacterial taxa (e.g., *Arthrobacter* sp., *Paenibacillus* sp., and *Carnobacterium* sp.) in samples collected from higher altitudes. Alpha diversity was also significantly lower in skin samples collected from individuals living at higher altitudes. This suggests a potential future route to determine geolocation based on altitudinal parameters via the analysis of skin microbiome samples in the future—although here too, methodological refinement will be essential. Furthermore, understanding how skin microbiomes may fluctuate throughout the life course will also be an essential factor to consider.

Overall, all the models prioritize classification over prediction abilities. To enable real-time prediction of geographical coordinates from sampling data, increasing the sample sizes geographically and temporally, and developing more rigorous methods is essential.

### Personal Identification

A growing body of evidence suggests that human individuals may be uniquely identified based on stable autochthonous (i.e., native to a given environment) microbial profiles. This could have a substantial impact on forensic science—for example, in situations where the investigator cannot retrieve sufficient amounts of human DNA (i.e., from human somatic and germ cells). Yet it is unknown whether the variation in microbial communities between people is sufficient to identify individuals within large populations uniquely or stable enough to place them over time.

To answer some of these questions, Franzosa et al. (2015) tested different body site-specific microbial profiles and attempted to match them with 25–105 microbiome profiles during the person’s first and second visits to the sampling site. The authors reported that these profiles were useful in distinguishing individuals at the initial sampling time point and that 30% of the individuals were still uniquely identified several months later. In this study, gut microbiome samples were used to pinpoint 80% of individuals (*n* = 120) up to a year later. These results are encouraging—particularly in shorter timescales—however, they still suffer from relatively high variability. As such, greater improvements, e.g., in methods and sampling effort, will be needed before such approaches can be useful in a forensic setting.

High resolution melting analysis that targeted the 16S rRNA gene from oral swab samples have also been used to demonstrate its potential in distinguishing between individuals (Wang et al., 2019), albeit with a very small sample size in this study (*n* = 5). Schmedes et al. (2018) demonstrated accurate identification of individuals (*n* = 12) based on skin swab samples from different body sites (*n* = 14). They achieved 97% accuracy by sampling shirts and 96% accuracy using palm samples based on 1-nearest neighbor classification on nucleotide diversity of the bacterial genome. In another recent study, the researchers utilized a similar approach to identify individuals (*n* = 51). They analyzed microbiome samples collected from three different body sites—the manubrium (i.e., the upper-most segment of the sternum), the palmar surface of the hand, and the ball of the foot (Woerner et al., 2019). The researchers achieved 100% classification accuracy when conditioned on a maximum nearest neighbor distance for diversity, suggesting strong potential should these results be replicable in studies with much larger sample sizes.

Watanabe et al. (2018) suggested that minor taxa are one of the key factors for distinguishing between individuals. Their study analyzed microbiome samples (*n* = 66) from individuals (*n* = 11) over 2 years and achieved 85% accuracy in distinguishing individuals. They also used the same analytical methods to classify publicly available skin microbiome samples from individuals (*n* = 89) with a 78% identification accuracy. However, this level of accuracy is unlikely to be sufficient for forensic applications. The authors suggested that although personal identification is possible, the estimation of the accuracy decreases for larger cohorts due to increments of similar microbiome patterns. Overall, the use of microbiomics as a forensic tool to determine personal identification shows potential and technological viability and might be useful in situations where the investigator is unable to retrieve sufficient amounts of human DNA. Nonetheless, the findings fall short of the burden of proof. Improvements in the model’s sensitivity and specificity are required, and a methodology to address potential contamination issues. Furthermore, a better understanding of the microbial dynamics across time and space is essential for the findings to have a forensics value.

#### Biological Sex Determination

Recent evidence supports another contribution of microbiomics toward personal identification –the discrimination of biological sex, which could be useful where sufficient quantities of human DNA are unable to be retrieved. For example, airborne bacteria communities have previously been characterized in indoor environments (Chan et al., 2009). Luongo et al. (2017) investigated airborne bacterial and fungal diversity (i.e., constituents of the “aerobiome”) from different University dormitory rooms (*n* = 91). They used machine learning techniques and were able to predict the biological sex of room occupants with 79% accuracy based on relative abundances of the microbiota. Curiously, rooms occupied by males exhibited higher relative abundances of the microbiota. The authors suggested that it could be because males may shed more biological particles or use fewer cosmetic barriers such as skin lotions.

Biological sex-related differences in the human thanatomicrobiome—the microbial communities colonizing organs following death (*thanatos*, Greek for death) (Zhou and Bian, 2018)—have also been demonstrated by Bell et al. (2018). The authors compared amplicon signatures (using the 16S rRNA gene V1-2 and V4 regions) in the corpse heart tissue of 10 individuals and discovered key differences between males (*n* = 6) and females (*n* = 4). For example, *Streptococcus* sp. was found exclusively in male heart tissues, whereas females had a significantly higher prevalence of *Pseudomonas* sp. With refinement, such an approach could help to determine the biological sex of a corpse and the provenance of body parts.

In a study by Tridico et al. (2014), the authors “readily distinguished” male (*n* = 3) and female (*n* = 4) subjects based on the analysis of their pubic hair microbiomes. They identified *Lactobacillus* spp. that were unique to female participants. They also suggested that pubic hair is relatively insulated from the environment and colonized with niche-specific microbiota, which could be useful in forensic investigations. Unfortunately, the modest sample size of this study limits the conclusions that can be drawn from it. Nonetheless, the findings were supported by another small study by Williams and Gibson (2017), who identified individuals (*n* = 9) and their biological sex from pubic hair microbiota with an error ratio of 0.027 ± 0.058 and 0.017 ± 0.052, respectively. However, the sample sizes for all these studies are modest, and as such, further validation studies with larger sample sizes are needed before reliable conclusions can be drawn.

Interestingly, Phan et al. (2020) analyzed skin microbiome samples from both genders (*n* = 45) and found that the absence of the bacterial genus *Alloiococcus* could be useful in predicting female biological sex. The study showed a correlation between certain bacterial species and personal characteristics (e.g., biological sex). They specifically explored the presence/absence of microbiota from fingermarks left behind on surfaces and achieved a relatively modest 67% sex prediction accuracy using leave-one-out cross-validation analysis. Improvements in sample sizes and machine learning accuracy are necessary to explore the potential of this approach further. Additional research into whether certain bacteria (and other microorganisms) are distinct to females or simply related to external factors (such as cosmetic products on hands) would also be necessary.

#### Trace Evidence

There is an increasing interest in studying forensically relevant microbial profiles left behind on objects and surfaces. For instance, several studies showed that there is often a high level of bacterial presence on personal objects such as mobile phones (Koroglu et al., 2015; Kõljalg et al., 2017; Koscova et al., 2018; Kurli et al., 2018). Furthermore, human-associated items such as shoes and mobile phones have been shown to support distinct microbiomes (Lax et al., 2015; Coil et al., 2019).

Meadow et al. (2014) investigated the potential utility of mobile phones as “personal microbiome sensors.” They selected 17 individuals and collected three samples (the cell phone’s touch surface, their index finger, and their thumb). They demonstrated that bacterial communities sampled from mobile phones were more similar to their owners than other people. They found that about 22% of the taxa on participants’ fingers were also found on their phones, whereas only 17% were shared with other people’s phones. An individual’s index finger shared approximately 5% more OTUs with their mobile phone than with everyone else’s mobile phone in the study. Furthermore, 82% of the OTUs were shared between a person’s index finger and their phone. Although promising, here again, the sample size and accuracy of results need to be increased in future studies.

Kodama et al. (2019) found that postmortem skin microbiomes could be associated with personal objects with a high degree of accuracy. Several of the objects in the study were associated with 100% accuracy (i.e., medical devices, eyeglasses, bottles, and steering wheels), whereas objects like computer devices, remote controls, and cell phones were associated with over 67% accuracy, suggesting that with refinement, skin microbiome samples could be reliably linked to objects at the scene. Furthermore, studies have found that the postmortem skin microbiomes were stable and similar to antemortem skin microbiomes for up to 60 h postmortem (Kodama et al., 2019).

Salzmann et al. (2019) investigated the microbial profiles of different bodily fluids (*n* = 22). They identified source-specific microbial signatures from various bodily fluids. For example, the phyla *Proteobacteria* was associated with skin and semen sources, whereas *Firmicutes* showed a higher prevalence in saliva and vaginal secretions. Dobay et al. (2019) suggest that even when body fluid is exposed to indoor conditions for 30 days, samples continue to harbor body-site-specific microbial signatures. Hanssen et al. (2017) also demonstrated promising results, albeit with a small sample size (*n* = 6), for the microbially-mediated classification of body fluids. They performed pattern recognition by fitting a linear discriminant analysis model using Principal Component scores and were able to classify saliva samples in 94% of the cases.

Neckovic et al. (2020) recently investigated the potential transfer of skin microbiomes between individuals and substrates (i.e., allochthonous microbiota). They found that skin microbiota has been reliably transferred through direct contact, that is, between individuals shaking hands. Microbiota also transmits through indirect contact, as demonstrated by individuals rubbing a substrate and then swapping substrates with another person. The authors suggested that such analysis could be useful to corroborate sexual assault cases or other contact-related crimes. They also suggested that further research should consider the relative surface area of contact, pressure, friction, and the duration of the contact.

### Manner and Cause of Death

The ‘manner of death’ is a determination made by an expert following an investigation (e.g., a coroner, the police, or a medical examiner). Five manners of death are generally considered: natural, accidental, suicide, homicide, and undetermined (Advenier et al., 2016). Lutz et al. (2019) recently collected microbiome samples from 265 corpses from Finland, Italy, and the United States. The inspected cadavers differed in the manners of death: accidental death (*n* = 88), natural death (*n* = 106), homicide (*n* = 23), and suicide (*n* = 45). Their results suggested that *Lactobacillus, Enterobacteriaceae, Sediminibacterium, and Rhizobiales* were associated with different manners of death. With further research, these associations could be developed into predictive markers that help to determine the manner of death. However, as noted by the authors, *Sediminibacterium* and *Rhizobiales* bacteria may also represent environmental contamination, which needs to be controlled, and further validation through controlled experiments is needed to improve the reliability of their approach to determine the manner of death.

The potential of this microbiomics approach to determining the manner of death was corroborated in a recent study by Zhang et al. (2019) who, by obtaining samples during routine death investigations at the Wayne County Medical Examiner’s Office (Detroit, Michigan, United States), found different biomarkers associated with the manner of death. In this study, *Xanthomonadaceae* was more prevalent in cases related to hospital deaths, whereas *Actinomyces* sp. tended to be more prevalent in suicide cases. Increasing the numbers of samples generally increased the accuracy of the models. The authors cautioned that the prediction accuracy depends on the machine learning methods used and the number of anatomical sites analyzed. The authors suggest this study provides baseline information, and it could be possible to use machine learning to develop reference databases that allow microbially-mediated manner of death predictions in the future.

Kaszubinski et al. (2020) modeled beta-dispersion to test for manner and cause of death association using a microbiome data set of *n* = 188 postmortem cases (five body sites per case). The researchers demonstrated that beta-dispersion and demographic data could distinguish among manner and cause of death. In particular, they found that cardiovascular disease and drug-related deaths were correctly classified in 79% of cases. They found that binary logistic regression models were most effective at improving model success. This was an improvement over multinomial logistic regression models, which confirmed the manner and cause of death assessment only 62% of the time. The results of this study show promise for using postmortem microbiomes to indicate the manner of death. However, as the researchers’ highlight, sample sizes need to be greater. Moreover, the development of large databases will likely be required to train models with high success rates prior to being used in practical forensic contexts.

In terms of cause of death (i.e., the disease or injury that produces physiological disruption in the body leading to death), researchers such as Christoffersen (2015) have investigated the importance of microbiological testing. Studying autopsy results (*n* = 42), the author reported that the cause of death could be determined in 42% of the cases via microbiological analysis. The study highlighted factors indicative of a microbiologically related cause of death, such as a raised CRP measurements. Raised CRPs have also been implicated in SIDS as a cause of death (Rambaud et al., 1999; Szydlowski et al., 2013) and even for astronauts returning from space (Garrett-Bakelman et al., 2019). Deadly bacterial infections, such as infection or sepsis, may also occur following neonatal circumcision (Elhaik, 2016, 2019).

A specific forensic microbiome application for determining the cause of death is the diagnosis of ‘death by drowning,’ which is one of the leading causes of unnatural deaths worldwide (Domínguez et al., 2018; Cenderadewi et al., 2019). Analyzing the presence of diatoms (single-celled algae) has been the ‘gold standard’ for well over a decade; however, its reliability has been questioned (Kakizaki et al., 2009; Huys et al., 2012). Several studies have provided support for death by drowning diagnoses by designing real-time PCR assays with primers to detect bacterial species associated with aquatic environments, such as *Aeromonas* spp. (Aoyagi et al., 2009; Uchiyama et al., 2012; Rutty et al., 2015; Voloshynovych et al., 2019). These studies provided support for this cause of death diagnosis based on relatively high detection rates of microbiota, for example, 84% (*n* = 32), 75% (*n* = 20), and 84% (*n* = 43)—although to strengthen the cause of death diagnoses, the accuracy levels, and sample sizes could again be much improved. It has also been suggested that bioluminescent bacteria may be biomarkers for death by drowning in seawater. For example, Kakizaki et al. (2009) developed a simple assay targeting the 16S rRNA gene to identify bioluminescent colonies such as *Vibrio fischeri* and *Vibrio harveyi*. More recently, Lee et al. (2017) analyzed microbiome composition and pulmonary surfactant protein (SP-A) expression to develop a marker for diagnosis of death by drowning. They analyzed microbiota and histological appearance of both drowned and postmortem groups of experimental rats, comparing freshwater vs. marine water treatments. The authors found that 5513 and 5480 OTUs were unique to marine and freshwater, respectively. They also found that expression levels of SP-A were higher in the lungs of drowning victims compared to postmortem submersion. These findings could have important forensic value (e.g., determining both the type of environment and the timing of death) and demonstrate good potential for future applications. Marella et al. (2019) point out that other studies have focused on the presence of fecal bacteria, coliforms, and streptococcal bacteria to help determine the cause of death by drowning. These bacteria are sampled from the femoral artery and vein and the right and left ventricles. Fecal bacteria are considered to be always present in subjects who drowned compared with those with other cause of death diagnoses (Lucci et al., 2008; Marella et al., 2019). For example, Lucci and Cirnelli (2007) found fecal streptococcal presence in 100% of the freshwater drowning cases they studied (*n* = 22) and coliforms present in 90.91%. In this study, the control subjects (*n* = 30) uniformly showed an absence of fecal bacteria. In a later study, Lucci et al. (2008) assessed if the presence of these bacteria in the drowning medium could be detected in victims submerged after death. The researchers collected samples from drowned victims (*n* = 5 freshwater and *n* = 5 in seawater) and victims who were submerged after death (*n* = 3). Coliforms and streptococci were detected in all drowned victims but not in those submerged after death. These findings suggest that fecal coliforms and streptococci could be used as markers of drowning. However, the minuscule sample sizes must be interpreted with caution and increased considerably in future studies.

### Postmortem Interval

#### The Thanatomicrobiome

Determining the PMI (the time elapsed since a person has died) is often an essential part of a criminal investigation. To improve PMI prediction accuracy, researchers have begun examining the thanatomicrobiome (Javan et al., 2016; Burcham et al., 2019). Postmortem, these communities overwhelm the immune system allowing for subsequent colonization (Javan et al., 2019). Preliminary studies suggest that these microbial communities may undergo important successional changes in organs that could aid in determining the PMI (Adserias-Garriga et al., 2017).

Early studies on model animals suggest that this is feasible. For over a 48-day period of decomposition, Metcalf et al. (2013) aimed to uncover a “microbial clock” to provide an estimate of PMI by sequencing the 16S rRNA gene for bacterial and archaeal communities and the 18S rRNA gene for microeukaryotes. Their model provided reliable PMI estimates (±3 days) (*n* = 223). However, the study was conducted in controlled conditions using experimental mouse models—thereby necessitating a degree of caution when extrapolating the data to ‘real-life’ situations. Another study investigated the decomposition of pig cadavers. Their model predicted the PMI within 2–3 h of the time of death with 94.4% accuracy (Pechal et al., 2014), demonstrating promise with further methodological refinement. Pechal et al. (2018) carried out a large-scale study of body microbiome samples (*n* = 188) that found postmortem microbiomes were stable, reflecting antemortem microbiomes 24–48 h after death. The researchers also found that specific bacterial taxa were important in predicting health status. For example, *Haemophilus* and *Fusobacterium* were twice as abundant in healthy individuals, whereas *Rothia* was 0.09 times more abundant in heart disease cases. With further development, this could be used to indicate the state of human health during clinical investigations into a range of deaths, from chronic and natural to sudden and violent (Pechal et al., 2018). It is important to note that, although appropriate at the time, the bioinformatics approach used to process OTUs and to make functional predictions (e.g., QIIME 1.8 and PICRUSt 1) is now considered to be outdated. Furthermore, Amplicon Sequence Variants (ASV) may provide a richer taxonomic picture (Callahan et al., 2017).

Studying human subjects, Johnson et al. (2016) sampled the skin microbiome of decomposing human cadavers and developed an algorithm to estimate PMI. The authors achieved low error rates for skins samples and a PMI estimation accuracy of ±2 days (*n* = 144 from 21 cadavers), a substantial improvement compared to prior efforts (e.g., via entomological analysis). Belk et al. (2018) used 16S rRNA amplicon sequencing and found that creating models with the class or phylum taxonomic levels provided the most accurate predictions of PMI. This finding corroborated the study by Johnson et al. (2016) and illustrated its potential usefulness for forensics.

Another study using 454 pyrosequencing to determine abundances and diversity of the postmortem microbiome in several key organs such as the brain, heart, liver, and spleen found varying PMIs ranging from 29.5 to 240 h (Can et al., 2014). This study revealed that the most abundant taxa in postmortem microbial communities were the anaerobic, spore-forming Firmicute bacteria, *Clostridium* sp. Javan et al. (2017) confirmed that *Clostridium* sp. dominated at long PMIs, adding evidence to support the use of microbiomics in PMI determination in the future.

#### Localization Through Animal Microbiomes

Several studies have shown that animals from different taxonomic groups and environments possess unique microbial profiles. For example, Tibetan chickens *Gallus gallus*, Chinese Rhesus macaques *Macaca mulatta*, and plateau sheep *Ovis* spp. have unique gut microbiomes (Zhou et al., 2016; Huang et al., 2017; Zhao et al., 2018) shaped by genetic, geographical, and altitudinal factors. It has been demonstrated that the skin microbiomes of Estrildid finches, amphibians, bats, cetaceans, and dogs *Canis lupus familiaris* are unique (McKenzie et al., 2012; Avena et al., 2016; Erwin et al., 2017; Torres et al., 2017; Engel et al., 2018; Russo et al., 2018). Interestingly, Song et al. (2013) found that humans share microbial communities with their dogs.

With further investigations and methodological refinement, such capabilities point to the potential feasibility of linking a person with a site based on shared microorganisms with animals. Although further studies are needed, there is potential for forensic pathways to associate trace microbial profiles obtained from other species (unique to the given species) to a given environment and/or occupation (e.g., animal industries) or to pet ownership. For example, non-human animal-specific microbiota could potentially be detected on the body or clothing of a suspect or victim, which may be useful in the absence of sufficient animal DNA (i.e., from somatic and germ cells) evidence. This profile could then conceivably be traced to the point of contact with an animal or animal-based environments such as equine stables, pet shops, or zoos, thus complementing other traditional forensic evidence. However, this approach is mostly theoretical at the moment, and future research will be needed to test its feasibility.

## Discussion

As of today, microbiome-based forensics are almost absent from criminal investigations and courts. To explain why this is so, we may divide the different possible applications of microbial forensics into two groups: first, reconstruction issues such as the cause of death and PMI, which ask “what happened?” and help elucidate the circumstances of the crime; and second, comparison issues such as geolocation and personal identification, which ask “how similar are these two DNA profiles?” and may (dis)connect a suspect from an object or a place (e.g., murder weapon or crime scene). Reconstruction applications for forensic use are easier to develop since competing propositions are usually well-defined and limited in number; if sufficient research is invested in ascertaining the microbial characteristics associated with each combination of possible environmental, spatial and temporal conditions, then reconstruction becomes straight-forward. For example, if a cadaver is found buried at a depth of 1 m in a desert in summertime, and the temporal succession of the gut microbial community for these environmental conditions has previously been established, then PMI can be inferred with a high degree of accuracy and certainty. Thus, reconstruction microbiomics can readily pass the *Daubert* standard set by the US supreme court (Daubert v. Merrell Dow Pharmaceuticals Inc, 1993) to be recognized as admissible evidence bearing sufficient scientific foundation, including general acceptance in the scientific community, known and acceptable rate of error, and so on.

Comparison microbiomic tools, on the other hand, may provide greater benefit to the criminal investigation but are harder to develop to a level that would satisfy the *Daubert* standard. The most beneficial way to employ such tools would be in a “one-to-many” configuration, similar to forensic human DNA analysis: a DNA profile from trace evidence is compared to all the profiles (from known persons and locations) in a database, and if it exists in the database, its frequency in the relevant population (e.g., of soils) is calculated to enumerate the probability of encountering this profile by chance (i.e., originating from a location or person unrelated to the crime). At this time, however, there are hardly any relevant forensic databases of microbiomes that can be compared to trace evidence. In their absence, the only way to proceed in a forensic context is in a “one-to-one” configuration. For each criminal case, questioned samples (e.g., from a suspect’s shoe) are compared to context samples from the crime scene, alibi area, and other relevant sites. This approach provides less benefit to the investigation because it can only give conclusions of exclusion, that two samples do not share the same origin, or relative conclusions, such as “sample A is more similar to sample B than it is to sample C.” Inclusionary conclusions such as “sample A is very similar to sample B; the probability of a different sample, from another place, person or time, being this similar to sample B at random is 1 in X Million” is impossible without either an extensive database (which is costly to build and maintain) or thorough theoretical knowledge (which we do not have yet) regarding the factors that shape microbiomes. However, even for “one-to-one” analyses, we can provide a statistical evaluation of the evidence (Habtom et al., 2019) based on the Likelihood Ratio framework as recommended by the European Network of Forensic Science Institutes (Willis et al., 2015).

There are three more major hurdles to forensic tools of comparative microbiomics. The first one involves samples of mixed origins, containing substrates or DNA from different locations or persons. DNA analysis of mixtures is difficult even with simple human DNA profiles, and certainly more so with microbiomic DNA profiles which are far more complex. It is often impossible to tell with certainty which, or even how many, disparate microbiomes are present in the mixture, let alone accurately infer the DNA profile of each one. The famed 2016 report of the US President’s Council of Advisors on Science and Technology [President’s Council of Advisors on Science and Technology (US), 2016] found that the prevalent subjective analysis of complex human DNA mixtures by forensic experts is not a reliable methodology. Consequently, several computer programs were developed to interpret complex human DNA mixtures in an objective manner, and these are slowly being validated and accepted for routine forensic use. Software for objective analysis of microbiomic DNA mixtures may be built on this basis, but these are still years in the future. The second major hurdle is temporal variation. Contrary to human DNA, which can remain unchanged for years, microbial communities (both on the body and deposited as trace evidence) can fluctuate over time, often in correlation with changes in environmental parameters like moisture and pH (e.g., Pasternak et al., 2013). In cases where two samples for comparison are obtained at different times when markedly different environmental conditions prevail, mitigating the temporal changes in community structure is needed before analysis can ensue. So far, only a few studies have addressed this topic experimentally, mainly by applying various carbon sources to force the different microbial communities to “converge” (Pasternak et al., 2019), but so far with limited success. The third, and perhaps most challenging hurdle, is the problem of DNA transfer. In the past decade, human DNA evidence gained widespread credibility and acceptance in the courts so that the identification of a DNA profile from trace evidence as originating from a specific person is rarely disputed nowadays. Instead, it is becoming more and more common for the defense to challenge the method of deposition of the DNA, suggesting that it reached the crime scene by a legitimate activity (before or after the crime occurred) or by DNA transfer (e.g., when the innocent suspect shook the hand of the real perpetrator). This hurdle can sometimes be overcome by using the likelihood-ratio approach with activity-level propositions (Mayuoni-Kirshenbaum et al., 2020); however, similar to the former hurdles, this one is also very much still an open question, and it will take more time, effort, and research before microbiomics is ready to be employed and accepted within the legal system.

## Conclusion

Over the last decade, advances in genomic sequencing and bioinformatics have given rise to microbiomics, which fructified in a growing compendium of tools seeking to explore the panoply of microorganisms present in our bodies and environment. The evidence examined in this review indicates that microbiomics could be a forensically relevant and promising discipline with a multitude of applications—from determining substrate provenance and acquiring trace evidence to identifying individuals and estimating PMI. These advances may allow various microbiomic data, like those obtained from thanatomicrobiome analysis, to be used by forensic scientists to address questions related to criminal investigations, or at least be used alongside other forensic methods.

Throughout their life-course, humans and their microbiomes undergo complex interactions and co-adaptation processes involving nutrient intake and resulting in the production of decomposition products such as metabolites. Following a person’s death, these interactions change dramatically, and the microbiome composition and dynamics fluctuate accordingly. Understanding these colonizations and fluctuations represent major conceptual, methodological, and computational challenges—as do antemortem microbial dynamics. Related microbiome-based research in a forensics context and greater exploration of fungal and viral communities may also lead to an important enhancement in the forensic toolkit in the future.

Many challenges remain to overcome, such as contamination issues, modest study sample sizes, model over-specification and misspecification, prediction accuracies of machine learning techniques, understanding complex spatial and temporal variations in environmental microbiome dynamics, as well as risks and ethical concerns (Shamarina et al., 2017). Notably, even human DNA-based evidence, which is far better understood, is not error-proof, as indicated by the Phantom of Heilbronn case (Daniel and van Oorschot, 2011). Moreover, the vast majority of published work used 16S or targeted sequencing approaches, which have known limitation for taxonomic resolution and could likely benefit from metagenomics methods (Ogilvie and Jones, 2015; McIntyre et al., 2017) and/or methods that utilize longer reads (Danko et al., 2019b; Foox et al., 2020). Also, more field-based testing and deployment of these sequencing methods could benefit from rigorous, titrated standards for ensuring accuracy (McIntyre et al., 2019). Given this, many of the applications reported in the literature should be considered proof of concepts rather than full-fledged forensic applications. Nonetheless, as Ogilvie and Jones (2015, p. 1) have summarized: “*it is clear that we remain in a period of discovery and revelation, as new methods and technologies begin to provide [a] deeper understanding of the inherent ecological characteristics of this [microbial] ecosystem.”*

## Author Contributions

EE initiated the study. JR carried out the review. EE, JR, ZP, and CM wrote the manuscript. All authors approved the manuscript.

## Conflict of Interest

EE consults the DNA Diagnostics Center. CM is a co-founder of Biotia, Inc. The remaining authors declare that the research was conducted in the absence of any commercial or financial relationships that could be construed as a potential conflict of interest.

## Abbreviations

PCR: polymerase chain reaction
MetaSUB: metagenomics and metadesign of subways and urban biomes
PRISMA: preferred reporting items for systematic reviews and meta-analyses
CAMDA: critical assessment of massive data analysis
PMI: postmortem interval
OUT: operational taxonomic unit
CRP: C-reactive protein
SIDS: sudden infant death syndrome.
